# Supplementary material for: Comprehensive Metabolomics in Mouse Mast Cell Model of Allergic Rhinitis for Profiling, Modulation, Semiquantitative Analysis, and Pathway Analysis
Source: Biomolecules. 2025 Jan 11;15(1):109. doi: 10.3390/biom15010109 (PMC11763337; doi:10.3390/biom15010109)
Supplement: Supplementary file 1 [file biomolecules-15-00109-s001.zip › biomolecules-3382256-supplementary.pdf]

Comprehensive Metabolomics in Mouse Mast Cell Model of Allergic Rhinitis for Profiling, Modulation, Semiquantitative Analysis, and Pathway Analysis

Akshay Suresh Patil and Yan Xu *

Department of Chemistry, Cleveland State University, Cleveland, OH 44115, USA

*Corresponding author

Phone: +1-216-687-3991

Fax: +1-216-687-9298

Email address: y.xu@csuohio.edu

Table S1

The *p*-values and log2-fold-change values for the compared experimental conditions of the 44 regulated metabolites.

Metabolite name	C vs. S[a]			S vs. T[b]			S vs. Z[c]		
	p-value	Log2	Regulation[d]	*p*-value	Log2	Regulation	*p*-value	Log2	Regulation
(S)-beta-Methylindolepyruvate	*1.35×10^{-01}*	1.3	↑	**2.09×10^{-04}**	**2.5**	↓	**3.12×10^{-04}**	**5.1**	↑
2-Hydroxy-4-hydroxymethylbenzalpyruvate	*2.34×10^{-01}*	0.8	↓	**2.03×10^{-04}**	**3.8**	↓	**1.99×10^{-04}**	**4.2**	↓
3-Methylbutyl 2-oxopropanoate	*8.10×10^{-02}*	0.9	↓	**1.99×10^{-04}**	**2.6**	↓	**1.99×10^{-04}**	**5.0**	↓
8S,15S-diHPETE	**2.41×10^{-04}**	**8.5**	↑	**8.19×10^{-03}**	**2.7**	↓	**9.23×10^{-03}**	**4.2**	↓
9-HpETE	**2.76×10^{-02}**	**8.9**	↑	**4.27×10^{-03}**	**2.4**	↓	**9.56×10^{-03}**	**5.3**	↓
Aminofructose 6-phosphate	**1.99×10^{-04}**	**5.1**	↓	*6.78×10^{-01}*	1.0	↑	*4.91×10^{-01}*	0.8	↑
CerP(d18:1/20:0)	**5.00×10^{-02}**	**2.8**	↑	*1.86×10^{-01}*	0.8	↓	*2.19×10^{-01}*	0.2	↓
DHAP(18:0)	**2.05×10^{-04}**	**8.1**	↑	*1.06×10^{-01}*	1.2	↓	1.99×10^{-04}	0.3	↓
Dihydroxyacetone Phosphate Acyl Ester	*1.23×10^{-01}*	1.4	↑	**3.07×10^{-04}**	**2.1**	↑	**8.71×10^{-04}**	**9.0**	↑
Enol-phenylpyruvate	*1.95×10^{-01}*	1.2	↑	*1.53×10^{-01}*	1.0	↑	**3.02×10^{-04}**	**3.5**	↑
Ethyl pyruvate	**5.07×10^{-04}**	**2.3**	↑	**1.99×10^{-04}**	**2.9**	↓	*2.89×10^{-01}*	1.1	↑
Glutathione (GSH)	**6.96×10^{-04}**	**2.4**	↑	**1.77×10^{-02}**	**2.3**	↓	*1.28×10^{-01}*	0.1	↓
Oxiglutatione (GSSH)	**1.99×10^{-04}**	**5.2**	↑	**2.10×10^{-04}**	**3.2**	↓	*3.52×10^{-01}*	1.9	↓
Histamine	**2.42×10^{-04}**	**3.8**	↑	**4.95×10^{-03}**	**3.5**	↓	*5.07×10^{-01}*	0.2	↓
L-Arginine	**2.02×10^{-04}**	**6.2**	↑	**6.47×10^{-04}**	**4.1**	↓	**1.99×10^{-04}**	**5.2**	↓
L-Asparagine	**3.90×10^{-02}**	**4.6**	↑	**4.57×10^{-02}**	**2.3**	↓	*4.53×10^{-01}*	0.6	↓
L-Aspartic Acid	*3.48×10^{-01}*	1.3	↑	*4.82×10^{-01}*	0.3	↑	**2.79×10^{-03}**	**2.1**	↑
Leukotriene A4	**1.99×10^{-04}**	**9.0**	↑	**1.99×10^{-04}**	**2.4**	↓	**3.24×10^{-02}**	**2.3**	↓
Leukotriene D4	**3.48×10^{-02}**	**3.4**	↑	*2.27×10^{-01}*	1.1	↓	**4.38×10^{-04}**	**3.2**	↓
Leukotriene E4	**9.43×10^{-03}**	**5.0**	↑	*3.96×10^{-01}*	0.8	↓	**1.99×10^{-03}**	**4.9**	↓
L-Glutamine	**1.99×10^{-04}**	**3.2**	↑	**8.16×10^{-03}**	**3.8**	↓	**4.27×10^{-03}**	**3.2**	↓
L-Histidine	**1.48×10^{-02}**	**2.8**	↓	*1.62×10^{-01}*	1.3	↑	*1.79×10^{-01}*	1.2	↓
Lipoxin C4	**9.32×10^{-04}**	**2.1**	↓	*2.58×10^{-01}*	1.6	↓	*1.13×10^{-01}*	2.2	↓
L-Isoleucine	*5.58×10^{-01}*	1.5	↑	*5.31×10^{-01}*	1.2	↑	**2.10×10^{-04}**	**2.0**	↑
L-Phenylalanine	**1.99×10^{-04}**	**3.8**	↑	**3.05×10^{-02}**	**2.8**	↓	**1.99×10^{-04}**	**2.8**	↓

L-Proline	**5.36 x 10^{-04}**	**3.3**	↑	**2.00 x 10^{-04}**	**2.7**	↓	**1.04 x 10^{-02}**	**2.5**	↓
L-Serine	**5.72 x 10^{-04}**	**8.0**	↑	*4.41 x 10^{-01}*	2.5	↓	2.02 x 10^{-02}	0.9	↓
Leukotriene B3 (LTB3)	**1.99 x 10^{-04}**	**3.2**	↑	**1.53 x 10^{-02}**	**2.1**	↓	**2.32 x 10^{-02}**	**2.5**	↓
Leukotriene B4 (LTB4)	**3.86 x 10^{-04}**	**4.0**	↑	1.48 x 10^{-02}	1.8	↓	**6.47 x 10^{-04}**	**3.8**	↓
Leukotriene B5 (LTB5)	**2.05 x 10^{-04}**	**3.0**	↑	**3.27 x 10^{-02}**	**2.1**	↓	**3.01 x 10^{-03}**	**3.0**	↓
L-Tryptophan	*1.35 x 10^{-01}*	1.3	↑	*7.32 x 10^{-01}*	1.8	↑	**2.13 x 10^{-04}**	**3.0**	↑
L-Valine	**8.15 x 10^{-04}**	**4.3**	↑	**2.00 x 10^{-04}**	**2.7**	↓	**8.16 x 10^{-03}**	**3.0**	↓
Dinoprost (PGF2α)	**1.11 x 10^{-02}**	**3.4**	↑	**4.27 x 10^{-03}**	**2.5**	↓	**3.10 x 10^{-03}**	**2.6**	↓
Epoprostenol (PGI2)	**4.20 x 10^{-02}**	**3.1**	↑	**3.53 x 10^{-04}**	**2.4**	↓	**2.02 x 10^{-03}**	**2.4**	↓
S-(4-Bromophenyl)-mercaptopyruvate	*9.40 x 10^{-02}*	1.2	↑	*1.49 x 10^{-01}*	1.1	↓	**1.78 x 10^{-03}**	**3.0**	↓
Thromboxane	**9.50 x 10^{-03}**	**2.5**	↑	*2.09 x 10^{-01}*	1.1	↓	**3.10 x 10^{-03}**	**0.4**	↑
Thromboxane A2 (TXA2)	**2.41 x 10^{-04}**	**2.1**	↑	**2.04 x 10^{-03}**	**3.0**	↓	**2.05 x 10^{-03}**	**3.5**	↓
Thromboxane A3 (TXA3)	**1.99 x 10^{-04}**	**5.0**	↑	**2.37 x 10^{-04}**	**2.3**	↓	**5.58 x 10^{-03}**	**2.3**	↓
Thromboxane B1 (TXB1)	*4.15 x 10^{-01}*	6.3	↑	*9.60 x 10^{-02}*	5.6	↓	**1.03 x 10^{-02}**	**5.3**	↑
Thromboxane B2 (TXB2)	**1.99 x 10^{-04}**	**3.1**	↑	**2.40 x 10^{-03}**	**4.2**	↓	**1.33 x 10^{-03}**	**4.2**	↓
Retinoic acid	**2.33 x 10^{-03}**	**4.3**	↑	*6.22 x 10^{-01}*	1.5	↓	*3.17 x 10^{-01}*	1.4	↓
Cer(d18:1/16:0)	**3.32 x 10^{-03}**	**5.0**	↑	**4.18 x 10^{-02}**	**5.3**	↓	**1.42 x 10^{-03}**	**4.8**	↓
Cer(d18:1/14:0)	**1.39 x 10^{-03}**	**4.8**	↑	**4.18 x 10^{-02}**	**4.6**	↓	**2.04 x 10^{-03}**	**4.8**	↓
Arachidonic acid	**5.24 x 10^{-03}**	**4.6**	↑	**2.48 x 10^{-04}**	**3.9**	↓	**2.24 x 10^{-02}**	**3.8**	↓

[a] C vs S: Control vs. LPS-stimulated

[b] S vs T: LPS-stimulated vs. triprolidine-treated post-LPS stimulation

[c] S vs Z: LPS-stimulated vs. zileuton-treated post-LPS stimulation

[d] Regulation: Symbol "↑" stands for upregulation of metabolite in the comparison group and symbol "↓" stands for downregulation of metabolites in the comparison group

Note: The metabolic changes with *p* and log2-fold-change values highlighted in bold met the criteria of *p*-value < 0.05 and log2 FC > 2.0.
